# The effects of anodal transcranial direct current stimulation and patterned electrical stimulation on spinal inhibitory interneurons and motor function in patients with spinal cord injury

**DOI:** 10.1007/s00221-016-4561-4

**Published:** 2016-01-20

**Authors:** Tomofumi Yamaguchi, Toshiyuki Fujiwara, Yun-An Tsai, Shuen-Chang Tang, Michiyuki Kawakami, Katsuhiro Mizuno, Mitsuhiko Kodama, Yoshihisa Masakado, Meigen Liu

**Affiliations:** Department of Rehabilitation Medicine, Keio University School of Medicine, Shinjuku-ku, Tokyo, Japan; Department of Rehabilitation Medicine, Tokai University School of Medicine, 143 Shimokasuya, Isehara, Kanagawa 259-1193 Japan; Center for Neural Regeneration, Taipei Veterans General Hospital, Taipei, Taiwan, ROC; National Yang Ming University, Taipei, Taiwan, ROC

**Keywords:** H-reflex, Disynaptic reciprocal inhibition, Presynaptic inhibition, Spinal plasticity, Locomotion, Rehabilitation

## Abstract

Supraspinal excitability and sensory input may play an important role for the modulation of spinal inhibitory interneurons and functional recovery among patients with incomplete spinal cord injury (SCI). Here, we investigated the effects of anodal transcranial direct current stimulation (tDCS) combined with patterned electrical stimulation (PES) on spinal inhibitory interneurons in patients with chronic incomplete SCI and in healthy individuals. Eleven patients with incomplete SCI and ten healthy adults participated in a single-masked, sham-controlled crossover study. PES involved stimulating the common peroneal nerve with a train of ten 100 Hz pulses every 2 s for 20 min. Anodal tDCS (1 mA) was simultaneously applied to the primary motor cortex that controls the tibialis anterior muscle. We measured reciprocal inhibition and presynaptic inhibition of a soleus H-reflex by stimulating the common peroneal nerve prior to tibial nerve stimulation, which elicits the H-reflex. The inhibition was assessed before, immediately after, 10 min after and 20 min after the stimulation. Compared with baseline, simultaneous application of anodal tDCS with PES significantly increased changes in disynaptic reciprocal inhibition and long-latency presynaptic inhibition in both healthy and SCI groups for at least 20 min after the stimulation (all, *p* < 0.001). In patients with incomplete SCI, anodal tDCS with PES significantly increased the number of ankle movements in 10 s at 20 min after the stimulation (*p* = 0.004). In conclusion, anodal tDCS combined with PES could induce spinal plasticity and improve ankle movement in patients with incomplete SCI.

## Introduction

Spinal inhibitory reflexes mediated by gamma-aminobutyric acid (GABA) and glycine are often absent or reduced in patients with spinal cord injury (SCI) (Calancie et al. 1993; Okuma et al. 2002). This reduced spinal reciprocal inhibition is thought to contribute to poor movement ability and abnormal muscle coactivation during locomotion (Fung and Barbeau 1989; Knikou and Mummidisetty 2011; Bhagchandani and Schindler-Ivens 2012).

Rehabilitation training can induce some plastic changes in spinal reflex circuits and help the recovery of lower limb function following SCI (Knikou and Mummidisetty 2014; Knikou et al. 2015). However, this plastic change and recovery are often limited (Raineteau and Schwab 2001; Thompson et al. 2006, 2009). Strategies for further enhancing spinal plasticity and improving lower limb function in individuals with SCI are sorely needed. One such strategy is patterned electrical stimulation (PES) that is applied to the common peroneal nerve and mimics input from primary ankle-dorsiflexor afferents during walking. This stimulation normally produces a short burst of firing at the beginning of the swing phase during stepping, and induces short-term reciprocal inhibition (RI) plasticity in healthy individuals (Perez et al. 2003).

Supraspinal modulation is thought to play an important role in inducing long-lasting spinal plasticity (Wolpaw and Tennissen 2001; Chen et al. 2006; Fujiwara et al. 2011; Yamaguchi et al. 2013), which may contribute to the recovery of limb and walking function following SCI (Knikou and Mummidisetty 2014; Knikou et al. 2015; Kaegi et al. 2002; Chen et al. 2006, 2010). Anodal transcranial direct current stimulation (tDCS) is a non-invasive form of artificial supraspinal modulation that stimulates brain regions by delivering weak direct currents through the skull, and can increase motor cortex excitability in both healthy individuals and neurological patients (Nitsche and Paulus 2000; Suzuki et al. 2012).

Fujiwara et al. (2011) showed that applying tDCS before PES modulated the effects of PES on RI in a polarity specific manner. Supraspinal modulation may be able to strengthen RI changes with PES. Therefore, we hypothesize that combining anodal tDCS to the primary motor cortex (M1) with PES may enhance the plastic changes in RI induced by PES in patients with incomplete SCI, who lack descending cortical drive to the spinal cord. Furthermore, these changes may result in improved ankle movements in these patients. The objective of our study was to exam the effects of anodal tDCS combined with PES on RI and ankle movements in patients with incomplete SCI.

## Materials and methods

The study protocol was approved by the Committee of Medical Ethics of National Yang Ming University, Taiwan, and written informed consent was obtained from all participants. All procedures complied with the Declaration of Helsinki.

### Participants

#### Healthy participants

Ten healthy volunteers (9 males) aged 35–65 years (mean ± SD, 50.7 ± 8.9 years) participated in this study. None of the participants had a history of neurological disease or were receiving any acute or chronic medication affecting the central nervous system.

#### Patients with spinal cord injury

Eleven patients who met the following criteria participated in the study of the 58 inpatients and 110 outpatients with incomplete SCI: (1) aged 20–65 years; (2) incomplete SCI as classified by a rank of C or D on the American Spinal Cord Injury Association Impairment scale (Maynard et al. 1997); (3) the SCI occurred more than 180 days before the experiment; (4) ankle plantar-flexor spasticity was at least 1+ on the modified Ashworth scale (Bohannon and Smith 1987); (5) ankle dorsiflexion had a manual muscle testing grade >1; (6) passive range of motion (ROM) at the ankle joint was ≥10° for dorsiflexion and ≥20° for planter flexion. No patient was receiving antispastic medication or other drugs that could interfere with cortical excitability. Demographic and clinical characteristics of the patients are summarized in Table 1. The mean age ± SD was 51.8 ± 10.7 years (range 28–64 years), and the mean time since SCI ± SD was 4.5 ± 4.2 years (range 0.6–12.1 years).

**Table 1:** Profiles of patients with spinal cord injury

	Age (years)	Gender	Time since SCI (month)	Cause	Dominant affected side	ASIA impairment scale		Neurological level	MAS	WISCI
						Motor level	Strength grade (TA/SOL)			
1	52	Male	8	T	Left	C	2/2	C4	1+	0
2	64	Male	12	T	Right	D	1/1	C1	3	4
3	38	Male	20	NT	Left	D	1/1	T4	1+	6
4	52	Male	7	NT	Right	D	2/2	T11	2	14
5	28	Male	52	T	Right	D	1/1	C4	2	8
6	63	Male	144	NT	Left	D	2/2	T11	1+	20
7	58	Male	30	T	Left	D	2/2	C4	2	13
8	49	Male	78	T	Left	D	2/2	C4	2	13
9	53	Male	59	T	Right	C	2/2	C5	2	0
10	59	Male	38	T	Right	D	2/2	C5	1+	14
11	54	Male	145	T	Right	D	1/2	C7	3	9

*T* trauma, *NT* non-trauma, *SCI level* the highest spinal cord level that was damaged, *ASIA* American spinal injury association impairment scale, *TA* tibialis anterior muscle, *SOL* soleus muscles, *MAS* modified ashworth scale, *WISCI* walking index for spinal cord injury

### Patterned electrical stimulation (PES)

We applied electrical stimulation to the common peroneal nerve (CPN) with a train of ten pulses (pulse duration, 1 ms) at 100 Hz every 2 s for 20 min (Perez et al. 2003). The stimulus intensity was set at the motor threshold of the tibialis anterior muscle (TA), without producing movement of the foot. The motor threshold for electrical stimulation was defined as the intensity that evoked a 100 μV response in the resting TA.

### Transcranial direct current stimulation (tDCS)

Anodal tDCS (1 mA, 20 min) was delivered by a DC STIMULATOR PLUS (NeuroConn, Ilmenau, Germany) connected to a pair of sponge-surface electrodes. The anodal electrode (surface area, 35 cm^2^) was positioned over the M1 contralateral to the TA of interest and the cathodal electrode (surface area, 50 cm^2^) was placed over the forehead above the ipsilateral orbit of the TA of interest. The position of M1 was confirmed based on induction of the largest motor evoked potentials in the TA with a constant stimulus intensity using transcranial magnetic stimulation (TMS) with a double-cone stimulation coil connected to a Magstim 200 magnetic stimulator (Magstim, Whitland, UK). Anodal and sham tDCS were administered in a random sequence to each participant, and were separated from each other by more than 3 days to minimize carry-over effects. For sham stimulation, the same procedure was used, but the current was delivered for only approximately 15 s to mimic the transient skin sensation felt at the beginning of the stimulation.

### Experimental paradigm

**Fig. 1:**
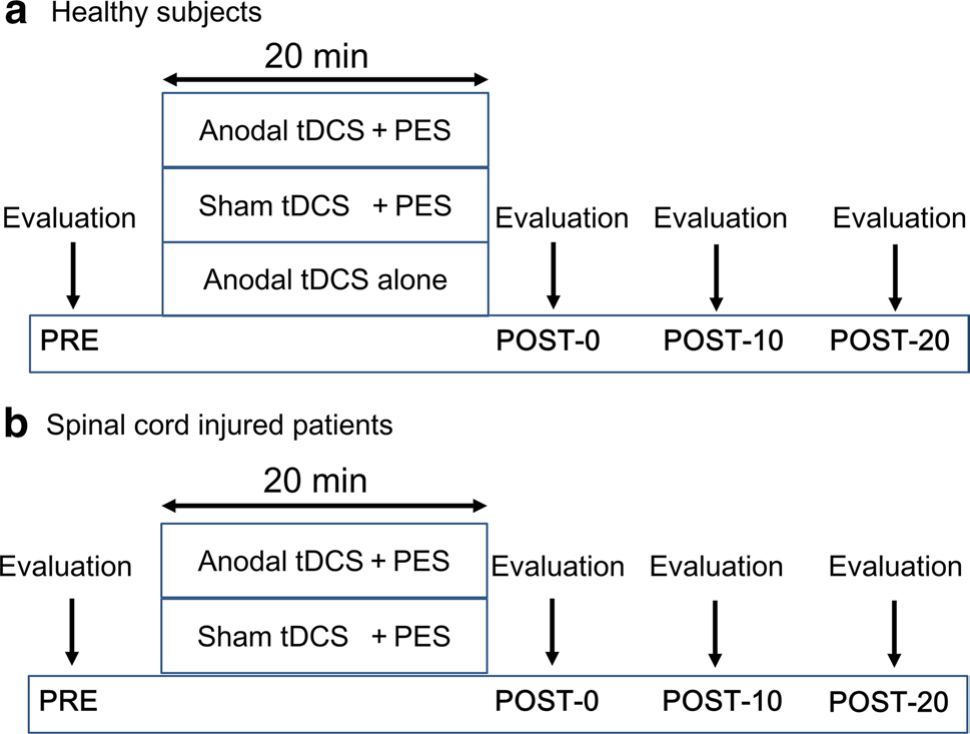
Experimental procedure. **a** Healthy individuals participated in the following three sessions: (1) anodal tDCS + PES; (2) sham tDCS + PES; (3) anodal tDCS alone. We measured the soleus H-reflex at baseline (PRE), immediately after (post-0), 10 min after (post-10), and 20 min after (post-20) the stimulation. **(b)** Patients with incomplete SCI participated in the following two sessions: (1) anodal tDCS + PES; (2) sham tDCS + PES. In patients with SCI, ankle movement was assessed before and 20 min after stimulation

The study employed a randomized, single-masked, crossover, sham-controlled experimental design. In all experiments, RI was measured before, immediately after, 10 min after, and 20 min after the intervention. Healthy individuals participated in the following three randomly-assigned sessions on three different days: (1) anodal tDCS combined with PES (anodal tDCS + PES); (2) sham tDCS combined with PES (sham tDCS + PES); (3) anodal tDCS alone (Fig. 1a). Patients with incomplete SCI participated in the following two sessions, randomly applied on separate days: (1) anodal tDCS + PES; (2) sham tDCS + PES (Fig. 1b). A computer generated list randomly assigned the order of the three or two paradigms. To washout the effects of each intervention, we set the interval at over 3 days prior to crossover. All participants lay supine with ankle joints set at 20° of plantar flexion and knee joints at 30° of flexion supported by a pillow. During the experiment, the head and arms were fully supported and participants were instructed to relax. Additionally, we carefully checked electromyograms for the soleus muscle (SOL) and TA to confirm the resting state of these muscles.

### Reciprocal inhibition

RI was assessed using a soleus H-reflex conditioning-test paradigm. The conditioning-test inter-stimulus interval (ISI) was set at 2, 20, or 100 ms to trigger inhibition through separate mechanisms (Mizuno et al. 1971). Inhibition at an ISI of 2 ms is called disynaptic reciprocal inhibition (RI_2ms_), and is mediated by a spinal glycinergic disynaptic inhibitory pathway. An ISI of 20 ms (RI_20ms_) is thought to result from presynaptic inhibition of the Ia afferent fibers that mediate the H-reflex. The origin of the inhibition at an ISI of 100 ms (RI_100ms_) is less clear, and may be attributed to presynaptic inhibition, modulated through long-loop inhibitory connections beyond the spinal cord (Mizuno et al. 1971; Huang et al. 2009). Ten conditioned and ten test H-reflexes were averaged at each time point: before (baseline), immediately after, 10 min after, and 20 min after the stimulation was completed. The H-reflex was elicited by stimulating the posterior tibial nerve at the popliteal fossa (1 ms rectangular pulse) with an anode on the patella. Throughout the experiment, the test H-reflex amplitude was maintained at 15–20 % of the amplitude of the maximum motor response for the SOL (Crone et al. 1990). Conditioning stimulation to the CPN was delivered using surface electrodes positioned below the fibular head. The conditioning stimulus strength was set at the motor threshold of the TA muscle. Motor threshold was defined as a 100 μV response measured at the TA. The CPN-stimulating electrode was carefully positioned to avoid activation of peroneus muscles, thus ensuring a more selective stimulation of the deep branch of the peroneal nerve. The conditioning stimulation was repeatedly checked during the experiments by monitoring the M wave from the TA muscle. The optimal interval for stimulating the CPN to produce the inhibition of RI_2ms_ was checked at an ISI of 0, 1 and 2 ms at the beginning of each session and used throughout. Conditioning-test intervals were tested in a random block design.

### Ankle movement

We measured ankle movement before and 20 min after the stimulation. Ankle movement was monitored by a video camera to count the number of ankle movements during a 10 s period, and we calculated the maximum range of motion. Patients were instructed to perform dorsi/plantar-flexion of the ankle as quickly as possible and with as wide a range as possible.

### Statistical analyses

For healthy persons, we applied a two-factor repeated-measures ANOVA to investigate the effects of paradigm (anodal tDCS + PES, sham tDCS + PES, and anodal tDCS alone) and time (before, immediately after, 10 min after, and 20 min after) on RI. For SCI patients, we used a two-factor repeated-measures ANOVA with paradigm (anodal tDCS + PES, sham tDCS + PES) and time (before, immediately after, 10 min after, and 20 min after) as factors. As for ankle movement, two-factor ANOVA, assessing paradigm (anodal tDCS + PES, sham tDCS + PES) and time (before and 20 min after) was used to analyze how the stimulation paradigm affected the number of ankle movements and range of motion. We performed a Pearson’s correlation analysis between the change in RI and the change in the number of ankle movements. The changes of RI and the number of ankle movements were calculated by subtracting the baseline data from data obtained 20 min after the stimulation was completed. Two sample *t* tests were performed to compare the baseline differences in RI between incomplete SCI patients and healthy participants. We applied a two-factor ANCOVA with group (healthy persons and SCI patients) and time (before, immediately after, 10 min after, and 20 min after) as factors to compare the effects of anodal tDCS + PES on RI between patients and healthy participants. For a two-factor ANOVA, post hoc analysis was performed using the paired *t* test to detect significant differences within and between paradigms in each time point. In an ANCOVA, the student’s *t* test was used to detect significant differences between group means after adjusting for the effects of RI baseline as a covariate. For these analyses, multiple comparisons with Bonferroni’s corrections were applied to adjust the *p* value for multiple comparisons (significance was set at 0.00185 because we compared 27 factors). Otherwise, *p* < 0.05 was considered significant. Statistical analyses were performed using IBM SPSS 22.0 (IBM Corp., Armonk, NY, USA) for Windows.

## Results

### Healthy participants

#### Reciprocal inhibition

The values of reciprocal inhibition are shown in Table 2. One-factor repeated-measure ANOVA showed no significant main effect of time (before, immediately after, 10 min after, or 20 min after) on test-H amplitudes under any stimulation paradigm (anodal tDCS + PES: *F* = 1.55, *p* = 0.224; sham tDCS + PES: *F* = 1.09, *p* = 0.370; anodal tDCS alone: *F* = 0.66, *p* = 0.582).

**Table 2:** Values of reciprocal inhibition

	RI_2ms_				RI_20ms_				RI_100ms_			
	Pre	Post	Post10	Post20	Pre	Post	Post10	Post20	Pre	Post	Post10	Post20
Healthy participants												
Anodal tDCS + PES	0.85 (0.06)	0.66** (0.09)	0.69** (0.06)	0.75** (0.04)	0.76 (0.09)	0.76 (0.08)	0.73 (0.10)	0.77 (0.07)	0.72 (0.12)	0.55** (0.15)	0.54** (0.18)	0.58** (0.16)
Sham tDCS + PES	0.86 (0.04)	0.70** (0.11)	0.77** (0.08)	0.86 (0.05)	0.80 (0.09)	0.82 (0.09)	0.77 (0.10)	0.77 (0.11)	0.74 (0.11)	0.73 (0.14)	0.71 (0.12)	0.77 (0.10)
Anodal tDCS alone	0.84 (0.08)	0.83 (0.07)	0.83 (0.09)	0.84 (0.07)	0.79 (0.10)	0.83 (0.10)	0.81 (0.09)	0.81 (0.09)	0.74 (0.08)	0.78 (0.14)	0.72 (0.14)	0.74 (0.11)
SCI patients												
Anodal tDCS + PES	1.17 (0.20)	0.80** (0.12)	0.85** (0.14)	0.93** (0.11)	1.07 (0.15)	0.93 (0.08)	0.91 (0.10)	0.99 (0.10)	1.08 (0.13)	0.74** (0.21)	0.79** (0.14)	0.85** (0.11)
Sham tDCS + PES	1.11 (0.21)	0.96* (0.11)	1.03 (0.13)	1.15 (0.19)	1.06 (0.23)	1.00 (0.23)	1.03 (0.17)	1.04 (0.19)	1.11 (0.17)	1.03 (0.17)	1.06 (0.16)	1.10 (0.22)

Data are presented as the mean ± SD. RI_2ms_ disynaptic reciprocal inhibition, RI_20ms_ D1 inhibition, RI_100ms_ D2 inhibition. Asterisks indicate that the differences in RI before and after stimulation were significant, as assessed by post hoc Bonferroni correction (* *p* < 0.05; ** *p* < 0.01)

At the baseline, RI_2ms_ mean (SD) were 0.85 (0.06), 0.86 (0.04) and 0.84 (0.08) with anodal tDCS + PES, sham tDCS + PES and anodal tDCS alone, respectively. The RI_20ms_ were 0.76 (0.09) with anodal tDCS + PES, 0.80 (0.09) with sham tDCS + PES, and 0.79 (0.10) with anodal tDCS alone. For the RI_100ms_ on baseline, anodal tDCS + PES was 0.72 (0.13), sham tDCS + PES was 0.74 (0.11), and anodal tDCS alone was 0.74 (0.08). Baseline of RI were not significantly different among the three interventions (ANOVA, RI_2ms_: *F* = 0.47, *p* = 0.630; RI_20ms_: *F* = 4.72, *p* = 0.629; RI_100ms_: *F* = 0.67, *p* = 0.935).

We found a significant interaction between paradigm (anodal tDCS + PES, sham tDCS + PES, anodal tDCS alone) and time (before, immediately after, 10 min after, and 20 min after) in RI_2ms_ (*F* = 9.12, *p* < 0.001) and RI_100ms_ (*F* = 10.37, *p* < 0.001), but not in RI_20ms_ (*F* = 1.41, *p* = 0.228). Post-hoc testing showed that RI_2ms_ and RI_100ms_ at baseline significantly differed from those at immediately after, 10 min after, and 20 min after anodal tDCS + PES. Post-hoc paired t test also showed that RI_2ms_ at baseline significantly differed from immediately after and 10 min after sham tDCS + PES. RI did not change at any time point when it was not accompanied by PES. Compared with anodal tDCS alone and sham tDCS + PES, changes in RI_2ms_ and RI_100ms_ were significant at immediately after, 10 min after, and 20 min after anodal tDCS + PES.

### Incomplete spinal cord injury patients

#### Reciprocal inhibition

**Fig. 2:**
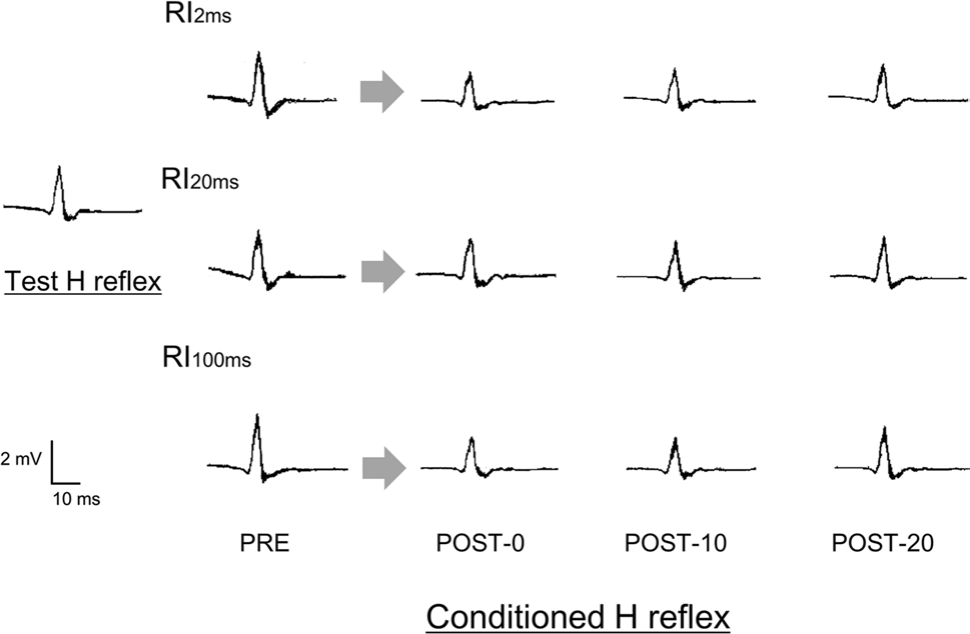
An example of test and conditioned H-reflex before and after anodal tDCS combined with PES in a single SCI patient. *Left* Test and conditioned H-reflex wave forms at baseline. *Right* Wave forms for the conditioned H-reflex immediately after (post-0), 10 min after (post-10), and 20 min after (post-20) anodal tDCS combined with PES. *Top* RI_2ms_, *Middle* RI_20ms_, *Bottom* RI_100ms_. RI_2ms_ mean conditioned H reflex amplitude at ISI 2 ms/mean test H reflex amplitude; RI_20ms_ mean conditioned H reflex amplitude at ISI 20 ms/mean test H reflex amplitude; RI_100ms_ mean conditioned H reflex amplitude at ISI 100 ms/mean test H reflex amplitude

The values of reciprocal inhibition are shown in Table 2. Figure 2 shows examples of how soleus H-reflex RI was modulated in individual participants. One-factor repeated-measures ANOVA showed no significant main effect of time (before, immediately after, 10 min after, or 20 min after) on the test-H amplitudes in anodal tDCS + PES (*F* = 1.55, *p* = 0.222) and sham tDCS + PES (*F* = 1.76, *p* = 0.177).

Baseline RI did not significantly differ between anodal tDCS + PES and sham tDCS + PES at any ISI (paired *t* tests, RI_2ms_: *p* = 0.125, RI_20ms_: *p* = 0.901, and RI_100ms_: *p* = 0.517). The mean RI_2ms_ (SD) were 1.16 (0.19) before anodal tDCS + PES and 1.10 (0.19) before sham tDCS + PES. The baseline of RI_20ms_ were 1.08 (0.14) and 1.07 (0.21) with anodal tDCS + PES and sham tDCS + PES, respectively. For the RI_100ms_ on baseline, anodal tDCS + PES was 1.10 (0.13) and sham tDCS + PES was 1.13 (0.17).

A two-factor repeated-measures ANOVA showed a significant interaction between paradigms (anodal tDCS + PES, sham tDCS + PES) and time (before, immediately after, 10 min after, and 20 min after) in RI_2ms_ (*F* = 10.08, *p* < 0.001) and RI_100ms_ (*F* = 7.79, *p* = 0.001), but not in RI_20ms_ (*F* = 0.89, *p* = 0.458). Post-hoc paired *t* tests revealed that anodal tDCS + PES induced significant changes from baseline in RI_2ms_ and RI_100m_ immediately after, 10 min after, and 20 min after the stimulation (all, *p* < 0.001). In the sham tDCS + PES, we found a significant difference for RI_2ms_ only immediately after the stimulation (*p* < 0.005). Comparison of anodal tDCS + PES with sham tDCS + PES showed significant difference in RI_2ms_ and RI_100ms_ at immediately after, 10 min after, and 20 min after the stimulation.

#### Ankle movement

**Fig. 3:**
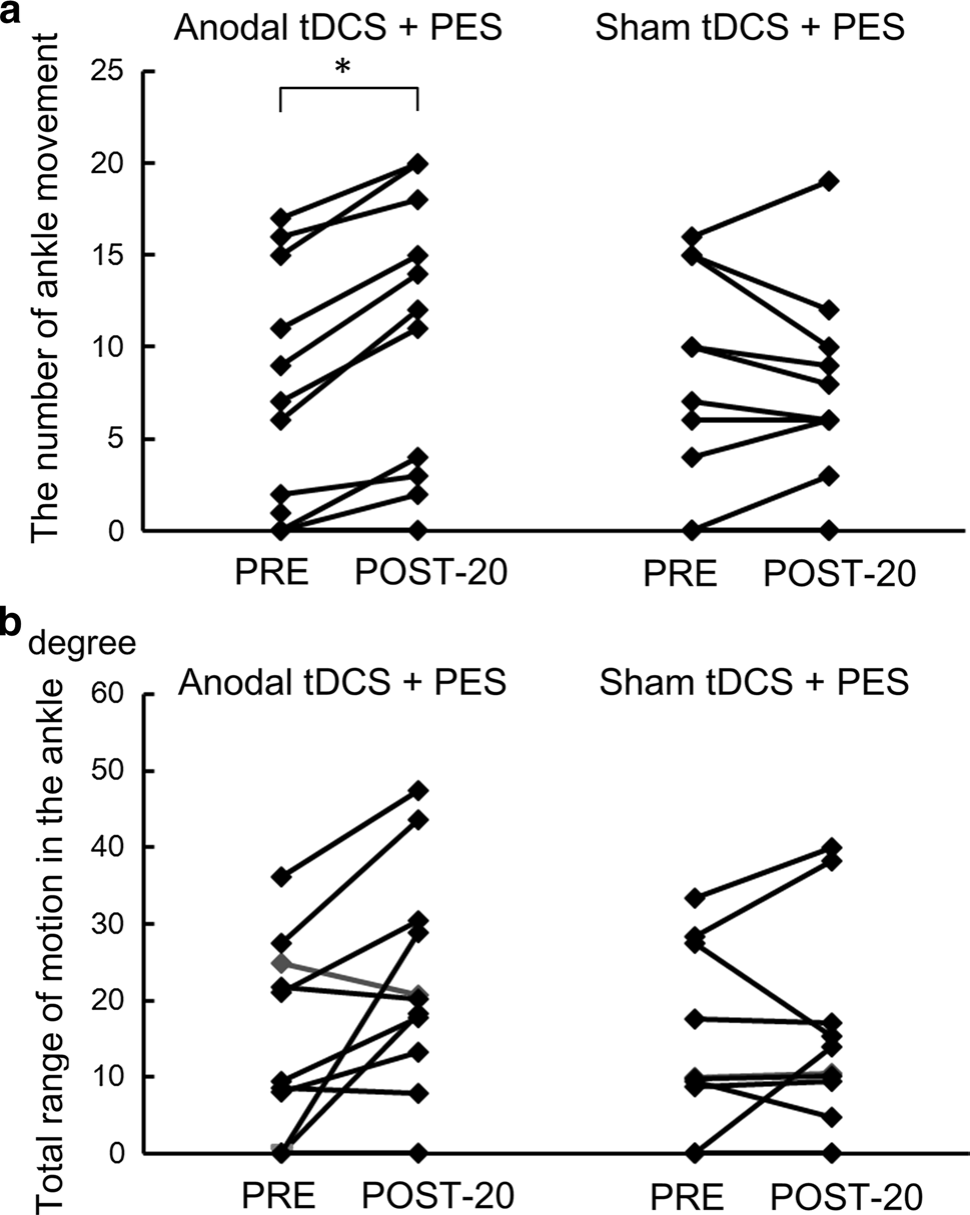
The effects of anodal tDCS (real and sham) combined with PES on the number and range of ankle movements. *Asterisks* show significant differences between baseline performance and that 20 min after the interventions (*p* < 0.05). **a** Effects of anodal tDCS + PES on the number of ankle movements for 10 s. **b** Effects of anodal tDCS + PES on the range of ankle movements

A two factor repeated measures ANOVA for the number of ankle movements showed a significant interaction of paradigm (anodal tDCS + PES and sham tDCS + PES) and time (before and 20 min after) (Fig. 3a; *F* = 13.65, *p* = 0.004), but not for the ankle range of motion (Fig. 3b; *F* = 4.89, *p* = 0.052). Pearson’s correlation analysis revealed significant negative relationships between the changes of RI_2ms_ and RI_100ms_, with the change in the number of ankle movements (RI_2ms_: *r* = −0.692, *p* = 0.018; RI_100ms_: *r* = −0.618, *p* = 0.043).

### The effects of anodal tDCS + PES on reciprocal inhibition in patients with incomplete SCI and in healthy participants

**Fig. 4:**
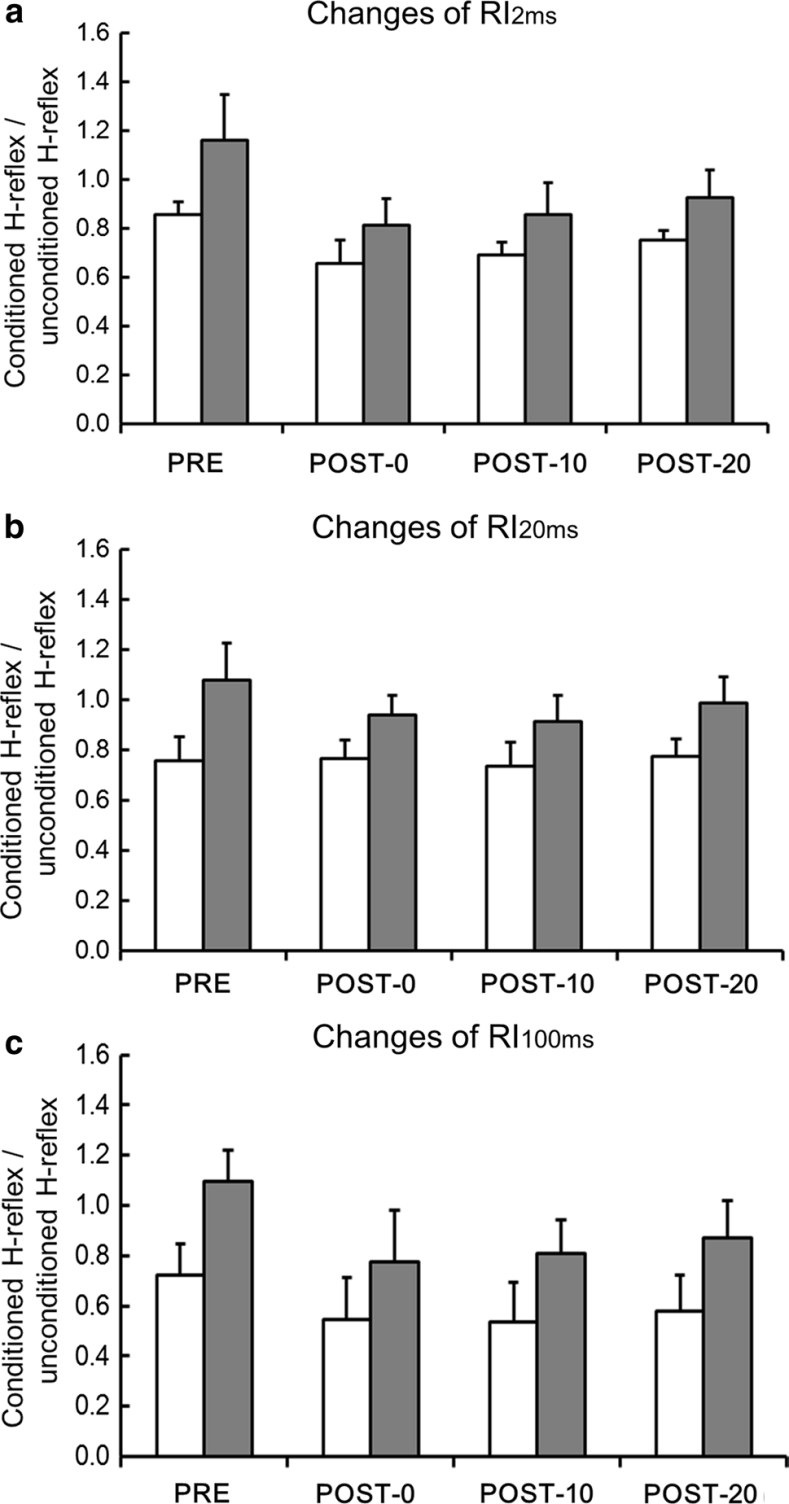
The effects of anodal tDCS + PES on RI in healthy persons and in patients with incomplete SCI. RI changes induced with anodal tDCS + PES in incomplete SCI were significantly different from that of the healthy-participant group (for both RI_2ms_ and RI_100ms_, *p* < 0.001), but not in RI_2ms_. **a** Effects of anodal tDCS + PES on RI_2ms_ in healthy persons (*white bar*) and in patients with incomplete SCI (*gray bar*). **b** Effects of anodal tDCS + PES on RI_20ms_ in healthy persons (*white bar*) and in patients with incomplete SCI (*gray bar*). **c** Effects of anodal tDCS + PES on RI_100ms_ in healthy persons (*white bar*) and in patients with incomplete SCI (*gray bar*)

The baseline of RI_2ms_, RI_20ms_, and RI_100ms_ were significantly higher in the incomplete-SCI group than in the healthy-participant group (*p* < 0.001). A two-factor ANCOVA, which included baseline (before intervention) as a covariate, showed a statistically significant main effect of group (incomplete-SCI group and healthy-participant group) on RI_2ms_ and RI_100ms_ (Fig. 4a: RI_2ms_: *F* = 8.29, *p* < 0.001, Fig. 4c; RI_100ms_: *F* = 10.15, *p* < 0.001), but not in RI_20ms_ (Fig. 4b: *F* = 2.09, *p* = 0.098). Post-hoc Student’s *t* test showed RI changes induced with anodal tDCS + PES in incomplete SCI were significantly different from that of the healthy-participant group (for both RI_2ms_ and RI_100ms_, *p* < 0.001).

## Discussion

We found that anodal tDCS combined with PES significantly decreased the value of RI_2ms_ and RI_100ms_, which means the magnitude of disynaptic reciprocal inhibition and long-latency presynaptic inhibition had increased in healthy participants and patients with chronic incomplete SCI. We also found a negative correlation between the changes in RI_2ms_ or RI_100ms_ and significant improvements in ankle movement at 20 min after the intervention in patients with chronic incomplete SCI. This result indicates that increased reciprocal inhibition improves ankle movement. Our findings provide the first evidence that plastic changes in disynaptic reciprocal inhibition and long-latency presynaptic inhibition are associated with improvements in ankle movement.

### Supraspinal modulation of reciprocal inhibition changes induced with PES

Anodal tDCS combined with PES increased the magnitude of disynaptic reciprocal inhibition and long-latency presynaptic inhibition for at least 20 min, while sham tDCS with PES increased the magnitude of disynaptic reciprocal inhibition immediately after in patients with SCI. Previous reports also showed that the change of disynaptic reciprocal inhibition induced with PES disappeared 10–20 min after the intervention in healthy persons (Perez et al. 2003; Fujiwara et al. 2011). This may mean that the benefits of plasticity on disynaptic reciprocal inhibition via PES are limited in terms of modulating impaired reflexes or spinal plasticity. Thus, simultaneously combining sensory input via PES with supraspinal excitation from anodal tDCS may be effective in inducing long-lasting spinal plasticity. Our results favor the hypothesis that the long-lasting change in disynaptic reciprocal inhibition that resulted from combining anodal tDCS with PES occurred because anodal tDCS increased the efficiency of the descending volleys reaching spinal inhibitory neurons. Studies have also indicated that Ia inhibitory interneurons in humans that project to soleus motoneurons might receive convergent input from motor areas in the brain and Ia afferents from the TA muscle (Nielsen et al. 1993; Masakado et al. 2001). Additionally, reports have shown that supraspinal modulation plays an important role in the induction and maintenance of longer lasting spinal plasticity (Chen et al. 2006; Fujiwara et al. 2011). For long-latency presynaptic inhibition, the change in inhibition was seen only after anodal tDCS combined PES, but not after sham tDCS combined PES. The physiological mechanism underlying changes in long-latency presynaptic inhibition under these circumstances is an unanswered question and should be examined in future studies. However, Huang et al. (2009) indicated that long-latency presynaptic inhibition was reduced after applying continuous theta-burst stimulation to the premotor cortex. They suggested that long-latency presynaptic inhibition is caused by long-loop inhibitory connections to supraspinal centers that receive input from the premotor cortex. In future studies, we should therefore examine activity in multiple brain areas (e.g., sensorimotor cortex, premotor cortex area) after combining anodal tDCS and PES using functional magnetic resonance imaging (fMRI). Our result, however, showed that combined anodal tDCS did not affect RI_20ms_, which means the magnitude of short-latency presynaptic inhibition was not changed by modulation of the motor cortex. Previous studies describe short-latency presynaptic inhibition is mediated through primary afferent depolarization (PAD) interneurons, which receive descending control from the brain stem rather than the primary motor cortex (Hongo et al. 1972; Rudomin and Schmidt 1999; Meunier and Pierrot-Deseilligny 1998). In addition, Roche et al. (2011) indicated short presynaptic inhibition did not change following application of anodal tDCS to the leg motor cortex in healthy individuals. Thus, it is possible that the increase of motor cortex excitability by applying anodal tDCS to the motor cortex may not modulate short-latency presynaptic inhibition.

### Reciprocal inhibition and ankle movement

Our results showed that the increase in magnitude of disynaptic reciprocal inhibition and long-latency presynaptic inhibition at 20 min after anodal tDCS combined with PES were correlated to improved ankle movement in patients with incomplete SCI. It was supposed that impaired spinal reciprocal inhibition induced gait or movement disturbances in patients with SCI, which is characterized by abnormal muscle contraction related to abnormal reflex modulation in ankle plantarflexion muscles (Dietz and Sinkjaer 2007; Nielsen et al. 2007). In our experiment, the increased magnitude of disynaptic reciprocal inhibition and long-loop presynaptic inhibition after anodal tDCS combined with PES are thought to have contributed to the improved quick turn needed for the dorsi-and plantar flexion of ankle movement. However, we believe that improvement of ankle movement could be explained by diminution of spasticity in plantar-flexor muscles. In future studies, spasticity should be examined using clinical assessments (i.e. modified Ashworth scale) by qualified clinicians at the different time points.

The increase in magnitude of disynaptic reciprocal inhibition and long-loop presynaptic inhibition induced with anodal tDCS combined with PES were greater in patients with SCI than in healthy participants. Some studies showed that RI of patients with SCI is impaired owing to loss of supraspinal input (Dietz 2002; Dietz and Sinkjaer 2007; Calancie et al. 1993; Okuma et al. 2002). It was supposed that the amount of reciprocal inhibition is related to the degree of functional motor recovery following SCI (Okuma et al. 2002). Therefore, the greater effect in patients was likely because of their injuries, which caused loss of descending cortical drive, which shows how important experiencing combined supraspinal excitability with sensory input is for promoting spinal plasticity and improving motor function in incomplete SCI patients.

### PES

The PES frequency was found on the primary afferents of ankle flexor muscles that produce a short burst of firing, with rates from 100 to 200 Hz at the beginning of the swing phase during stepping in animals (Prochazka and Gorassini 1998). PES can induce short-term RI plasticity (Perez et al. 2003). The detailed effects of stimulus parameters on RI plasticity remain largely unknown. Some studies indicate that peak firing frequencies of Ia inhibitory interneurons ranged from 50 to 300 Hz during rhythmic activity such as locomotion and scratching in animals (Feldman and Orlovsky 1975; Deliagina and Orlovsky 1980; Geertsen et al. 2011). Understanding the parameters that can best modify spinal networks is critical for optimizing rehabilitation strategies following SCI. Thus, in future studies, we should investigate more effective stimulus parameters that produce the largest inhibition and induce plasticity in RI.

### The effect of noninvasive brain stimulation on reciprocal inhibition

Another widely used noninvasive brain stimulation technique called repetitive transcranial magnetic stimulation (rTMS) was shown to reduce lower limb spasticity (Kumru et al. 2010), and restore impaired excitability in disynaptic reciprocal inhibition in subjects with SCI (Nardone et al. 2014). These reports support our hypothesis that increasing the excitability of the primary motor cortex using the modulation technique, such as anodal tDCS and high frequency rTMS, will increase descending input from the primary motor cortex to spinal inhibitory interneurons, which would then increase changes in disynaptic reciprocal inhibition and thus, improve ankle movement in patients with incomplete SCI.

In spite of the fact that rTMS has better spatial and temporal resolution, tDCS has some advantages over rTMS, such as its safer and easier application, and tDCS can be easily combined with other therapies (e.g. peripheral nerve electrical stimulation, or locomotion training), however, rTMS is difficult to accurately adjust. In particular, the combination of anodal tDCS and peripheral nerve electrical stimulation may have great potential for promoting spinal plasticity and motor recovery to expedite the rehabilitation process after central nervous system lesions (Celnik et al. 2009; Lindenberg et al. 2012). However, further studies are still needed to investigate whether the effects of anodal tDCS combined with PES on spasticity and gait function may induce more pronounced and beneficial clinical effects.

### Clinical application

Together with previous findings about the combined effects of anodal tDCS and peripheral nerve electrical stimulation on motor recovery after central nervous system injuries (Celnik et al. 2009; Lindenberg et al. 2012), our results suggest that the combination of anodal tDCS and PES might be an effective adjuvant therapy for functional recovery of locomotion. For example, it is possible to use this method in preparation for locomotor training such as treadmill walking with partial body weight support and robot-assisted locomotor training. This means that the spinal plasticity induced by anodal tDCS combined with PES may promote the effects of locomotor training on functional recovery of locomotion in patients with central nervous system injuries.

Present study, combining anodal tDCS with PES produced a restrictive effect on spinal plasticity of disynaptic reciprocal inhibition and long-loop presynaptic inhibition, suggesting that a single session itself might not be powerful enough for use in a clinical setting. Recently, daily application of tDCS was reported to have long-lasting beneficial effects on cognitive and motor functions (Lindenberg et al. 2012). These findings raise the possibility that repeated anodal tDCS combined with PES may have long-term beneficial effects on restoration of the spinal cord and motor function. In future studies, we would like to assess the long-term benefits of this technique in improving functional abnormalities and locomotion in the patient population.

## Conclusions

We found that combining anodal tDCS over the primary motor cortex and PES to the leg increased the magnitude of disynaptic reciprocal inhibition and long-loop presynaptic inhibition, and improved lower extremity motor function in patients with incomplete SCI. Although the strength of our conclusion must be tempered by the absence of data regarding the long-term effects on the restoration of the spinal cord and motor function, we provide the first evidence that anodal tDCS combined with PES is effective for improving impaired spinal modulation and recovery of motor function among patients with SCI. Therefore, therapy using this combination may be useful for the neuro-rehabilitation of patients with SCI.
